# Integrating impedance-based growth-rate monitoring into a microfluidic cell culture platform for live-cell microscopy

**DOI:** 10.1038/s41378-018-0006-5

**Published:** 2018-05-24

**Authors:** Ketki Chawla, Sebastian C. Bürgel, Gregor W. Schmidt, Hans-Michael Kaltenbach, Fabian Rudolf, Olivier Frey, Andreas Hierlemann

**Affiliations:** 1 ETH Zurich, Department of Biosystems Science and Engineering, Bio Engineering Laboratory, Basel, Switzerland; 2 ETH Zurich, Department of Biosystems Science and Engineering, Computational Systems Biology Group, Basel, Switzerland

## Abstract

Growth rate is a widely studied parameter for various cell-based biological studies. Growth rates of cell populations can be monitored in chemostats and micro-chemostats, where nutrients are continuously replenished. Here, we present an integrated microfluidic platform that enables long-term culturing of non-adherent cells as well as parallel and mutually independent continuous monitoring of (i) growth rates of cells by means of impedance measurements and of (ii) specific other cellular events by means of high-resolution optical or fluorescence microscopy. Yeast colonies were grown in a monolayer under culturing pads, which enabled high-resolution microscopy, as all cells were in the same focal plane. Upon cell growth and division, cells leaving the culturing area passed over a pair of electrodes and were counted through impedance measurements. The impedance data could then be used to directly determine the growth rates of the cells in the culturing area. The integration of multiple culturing chambers with sensing electrodes enabled multiplexed long-term monitoring of growth rates of different yeast strains in parallel. As a demonstration, we modulated the growth rates of engineered yeast strains using calcium. The results indicated that impedance measurements provide a label-free readout method to continuously monitor the changes in the growth rates of the cells without compromising high-resolution optical imaging of single cells.

## Cells: studying growth rates

Combining impedance-based measurements with real-time fluorescence microscopy in a microfluidic setup enables simultaneous observation of cellular processes and measurement of growth rates. Microfluidic devices are widely used to culture cells and monitoring their growth. Fluidic devices typically have been developed for either high-resolution imaging or for cell impedance characterization at high temporal resolution. Now, a team led by Andreas Hierlemann at ETH Zurich reports a setup, in which yeast colonies are grown in a monolayer – in a single focal plane – which enables high-resolution imaging of single cells and, at the same time, enables to acquire accurate growth-rate data through impedance measurements, as the colony grows . In addition, multiple culturing chambers permit to monitor different cell strains under various experimental conditions in parallel. Optical imaging and impedance measurement are fully decoupled allowing great readout flexibility.

## Introduction

Cells regulate their growth rate in response to external signals, and as cells grow, their metabolism, macromolecular synthesis, and the processes included in cell division must be coordinated^1–4^. This coordination of different processes, the way in which cells monitor their nutritional environment, how they integrate this information into the cell cycle, how they regulate their cell cycle, as well as whether and how these regulatory processes change during a cellular life cycle still include many open issues^5–7^. The investigation of these open issues requires a well-developed and broadly understood model system, such as budding and fission yeast^8,9^, and an experimental setup that can be used to perform such investigations. The chemostat provides a powerful method to systematically study the coupling between growth rates and cellular processes: it allows for experimentally controlling the growth rate of a cell population by adjusting the nutrient supply into a defined culture vessel volume, thereby providing a stable and defined environment for cells^10^. In a chemostat, the growth kinetics, i.e., the relation between cell growth rate and substrate consumption, is controlled by manipulating the medium addition to the culture vessel.

Micro-chemostats rely on microfluidics technology for culturing cells in a constant and defined environment under continuous perfusion. The cells in these devices grow in chambers or channels of defined size, and their growth rates are usually determined by using microscopy^11–15^. In contrast to conventional chemostats, the growth rates in these microfluidic platforms are defined by the composition of the supplied media. An advantage of microfluidic devices is that they do allow for monitoring of individual cells over an extended period of time. However, associated growth rate measurements are often limited by the field of view or the overall size of the culture chamber or pad and require dedicated software for cell segmentation and tracking. Detailed cell tracking requires high-temporal-resolution optical measurements, which limits the number of positions that can be imaged by the microscope in a single experiment due to the required stage movements. The limited number of imaging positions considerably reduces the throughput and detracts from the possibility to parallelize experiments under similar or identical conditions. Additionally, the use of fluorescence microscopy for measuring cell growth rates limits the number of fluorophores that are available for tracking other specific events and processes in the cells. Moreover, phototoxic effects may be induced upon frequent imaging^16^ so that additional control experiments become necessary to assess such phototoxicity effects, which are tedious to perform. Phototoxicity effects can be obviated by the use of label free techniques, such as measuring the optical density of the cell solution in microfluidic platforms^17,18^. Unfortunately, suitable devices are not amenable to high-resolution optical imaging and to obtaining information at single-cell resolution.

Electrical impedance spectroscopy (EIS) is a label free, non-invasive method for cell or particle counting and analysis^19–22^. Impedance cytometers, microfluidic devices with impedance measurement features offer the capability to characterize and analyze cell populations without the need for fluorescent labels^23–26^. A common implementation of microfluidic impedance platforms consists of simple microfluidic channels with single or multiple facing electrodes to perform the impedance measurements. Most of these flow-through platforms are stand-alone devices that can be used downstream of cell culture reactors or with cell suspensions, and are not easy to parallelize. Growth rate measurements in cell cultures using electrical cell-substrate impedance sensing (ECIS) were demonstrated for adherent cells^27,28^. Impedance-based measurements of viable biomass in microtiter plates were also performed for non-adherent cells^29,30^. However, to the best of our knowledge, there is currently no integrated platform, which features continuous and parallel execution of (i) cell culturing under controlled perfusion conditions, (ii) monitoring of the cell growth rate, and (iii) high-resolution imaging of non-adherent or suspended cells in the same single device.

Therefore, our goal was to develop a platform that allows for long-term culturing as well as continuous parallel and mutually independent monitoring of (i) growth rates by means of impedance measurements and of (ii) specific other cellular events by means of high-resolution optical or fluorescence microscopy. To this end, we used the concept of a previously developed microfluidic device to culture yeast cells in a defined volume underneath pads in a defined environment under continuous perfusion^31^. We then developed a new device, which, besides some other features, included an impedance readout to count the number of cells leaving culturing areas under the pads. This new microfluidic platform enables continuous impedance-based monitoring of the growth rates of populations of non-adherent cells, such as yeast cells, while it enables simultaneous long-term, high-resolution imaging of cellular events. Combined microscopy and impedance measurements can be conducted in an array-based format, as the device features multiple culturing chambers and sets of sensing electrodes. Multiple yeast cell colonies in different defined culturing environments can be analyzed in parallel. The setup is straightforward and simple to use on an automated microscope including all electrical connections for impedance measurements. Parallel growth rate measurements of different engineered yeast strains over 2 days have been conducted with the device. The growth rates of these strains were modulated by switching between standard medium and calcium-rich medium. The changes in the obtained growth rates were determined by impedance measurements and were similar to those observed by using standard culturing methods. In contrast to microscopy, impedance assessment enables high-throughput monitoring of growth rates without imposing additional strain on cells as a consequence of light exposure. The developed platform enables the user to employ the full repertoire of microscopy methods for tracking a wide range of cellular events, while the growth rate of the cells is continuously and independently monitored through an impedance readout.

## Materials and methods

### Device fabrication

The device consists of two parts: a poly(dimethylsiloxane) (PDMS) layer containing the microfluidic structures, and a glass slide with a patterned metal layer. The PDMS layer was cast from a mold, fabricated in a three-layer microfabrication process on a 4-inch silicon wafer (Figure S 1a). The first layer for the clamping pads was obtained through selective ion-beam etching of a silicon substrate using a photoresist mask. This process allows for precise adjustment of the height of the first layer, which defines the clamping gap below the pads to be 4 ± 0.3 µm. The next two layers for the cell culture chambers and for the microfluidic channel structures were fabricated by two SU-8 photolithography steps. SU-8 25 and SU-8 100 (MicroChem Co., USA) were sequentially spin-coated to obtain layers of 20 and 200 µm height; the layers were then exposed to UV light through transparency masks for cross-linking. The mold was silanized for 2 h with trichloro(1H,1H,2H,2H-perfluoro-octyl)silane (Sigma-Aldrich, Switzerland) in a vacuum desiccator. A mixture of silicone and curing agent (10:1 w/w, Sylgard^®^ 184, Dow Corning, Germany) was poured on the SU-8 mold and cured for 2 h at 80 °C. The PDMS was then peeled off the mold and cut into single chips. Holes were punched for the device inlets and outlets.

The electrodes were fabricated on 4-inch 500-µm- or 200-µm-thick glass wafers using a lift-off process (Figure S 1b). The wafer was spin-coated with lift-off resist (LOR3B, Microchem Corp., Newton, USA), followed by a positive photoresist (S1813, Rohm-Haas, Schwalbach, Germany) and patterned using photolithography. A sputtering process was applied to deposit a platinum film of 200 nm thickness on top of a 20-nm-thick W/Ti adhesion layer. Metal lift-off was carried out using mr-Rem 400 remover (Micro Resist Technology GmbH, Berlin, Germany). A 500-nm passivation layer of silicon nitride was then deposited on the fabricated metal layer using plasma-enhanced chemical vapor deposition. The passivation layer was removed in the areas of electrodes and contacts pads (using a S1813 positive resist mask) by means of reactive-ion etching. After fabrication, the glass wafer was diced into individual glass slides (70 mm × 32 mm).

### Cell loading and device assembly

The cell loading procedure is schematically shown in Figure S 2. The assembly and precise alignment of the structures in the PDMS layer and the electrodes on the glass slide was carried out during the cell loading procedure with a custom-made alignment tool (Figure S 2a). The PDMS layer and glass slide were placed on the top and bottom holder of the alignment tool. The glass slide was brought into close proximity to the PDMS layer without touching it. The PDMS structures were aligned with the electrodes on the glass (X, Y, and theta) by using micrometer screws and a hand-held microscope (Dino-Lite digital microscope, Netherlands). Once aligned, the top holder with the PDMS layer was removed and placed on the table with the culture chambers facing upward; 0.5 µl of cell suspension at a concentration of 3 × 10^7^ cells/ml was pipetted into each chamber. As PDMS is hydrophobic in nature, the liquid did not spread. The top holder was again placed on the alignment tool. The four cone-shaped pins ensured that the top holder re-centered at the same position as aligned before removal. The glass slide was then moved upward and brought in contact with the PDMS layer. The assembled device was afterward removed from the alignment tool.

### Setup

#### Impedance measurements

The experimental setup is shown in Figure S 3a. After cell loading, the device was placed in a custom-made PCB (115 mm × 75 mm), which was used to switch between the sensing electrodes of different analysis units in an automated way (Figure S 3b). The PCB was connected to the impedance spectroscope (HF2, Zurich Instruments AG, Switzerland) and to a custom-made, automated, multiplexed EIS (AMEIS)^32^ controller board, which controlled the digital signal to switch between analysis units and provided the interface to a PC. Custom-made software was used to select the analysis units and to program the switching protocol and recording duration. The sensing electrodes of selected analysis units were connected to the impedance spectroscope for defined recording durations. Each analysis unit included two electrodes—a stimulating and a recording electrode. An AC signal with an amplitude of 2 V and frequencies of 1.12 and 1.5 MHz was applied to the stimulating electrode. At the recording electrode, the current was transformed into a voltage using a trans-impedance amplifier (HF2TA, Zurich Instruments AG, Switzerland), which was then measured using the impedance spectroscope. The data was stored on the PC for later analysis. The phase signal of the output voltage was recorded and analyzed. The obtained data were bandpass-filtered using MATLAB (The MathWorks Inc., USA) using a frequency range of 0.1–30 Hz. The peaks were extracted from the filtered data by applying a threshold to minimize the number of false positives and false negatives. The threshold values varied, depending on the medium conditions and frequency used for recording. The threshold values ranged between 2 × 10^−3^–6 × 10^−3^ degrees for 1.5 MHz and 0.5 × 10^−3^–0.8 × 10^−3^ degrees for 1.12 MHz.

#### Microscopy

The PCB hosting the device was placed in a custom-made holder, which fits onto the stage of an automated, inverted microscope (Figure S 3b). The images were obtained using inverted microscopes (Olympus IX 81 and Nikon Ti Eclipse microscope) placed in an environmental control box, which maintained a stable temperature of 30 °C. Fluorescence images were captured on the Nikon microscope using a Nikon Plan Fluor 40× objective (NA 0.75, WD 0.66). The microscope was controlled using Youscope^28^, and offline image analysis was performed using ImageJ^33^ and CellX^34^. Syringe pumps (neMESYS, Cetoni GmbH, Germany) were connected to the microfluidic chip for media supply (flow rate 10 µl/min, 15 ml of medium is required for a 24-h experiment) and were controlled using Youscope.

Experiments to assess the medium exchange characteristics (Figure S 4) were carried out on the Olympus microscope, and images were taken every second with a CMOS camera (Hamamatsu ORCA Flash 4.0 camera). Syringes were filled with de-ionized water and a solution of Amaranth (4 mg/ml, Sigma-Aldrich, Switzerland) in de-ionized water. Switching between the different syringes was controlled using Youscope. The calculation of the average intensity of the images and further analysis was performed using ImageJ.

### Cell culture

*Saccharomyces cerevisiae* strains—v*ph1*∆ (BY4741, Euroscarf, FRY2033) and wild-type strain (BY4700, FRY1398) were used for the experiments to measure growth rate dynamics in different medium conditions. These strains were cultured overnight in shaking flasks with Yeast extract Peptone Dextrose (YPD) containing 1% yeast extract (BD Biosciences, Germany), 2% peptone (BD Biosciences), and 2% glucose. The overnight culture was diluted and grown for a few hours in YPD to have the cells in the exponential phase before loading them into the device. Cells were grown in YPD with 100 mM CaCl_2_ to observe changes in the growth rate. Yeast strains carrying Vph1-Citrine::KanMX (FRY 2011), Whi5-Citrine::KanMX (FRY 1935), and Cdc12-Citrine::KanMX (FRY 2145) fluorescent fusion protein constructs were used to perform fluorescence microscopy. These strains were tagged with Citrine fluorophore but at different locations in the cell and cultured in synthetic defined (SD) media.

Growth rate measurements in a plate reader (Infinite M200 PRO, Tecan Group Ltd., Switzerland) were carried out for v*ph1*∆ and wild-type strains in YPD and YPD with 100 mM CaCl_2_. The absorbance of the cells was recorded at 600 nm every 6 min over a period of 72 h at 30 °C. The growth rate of the cells was measured as an increase in the absorbance over time, and was extracted using MATLAB.

## Results and discussion

### Device design and function

**Fig. 1:**
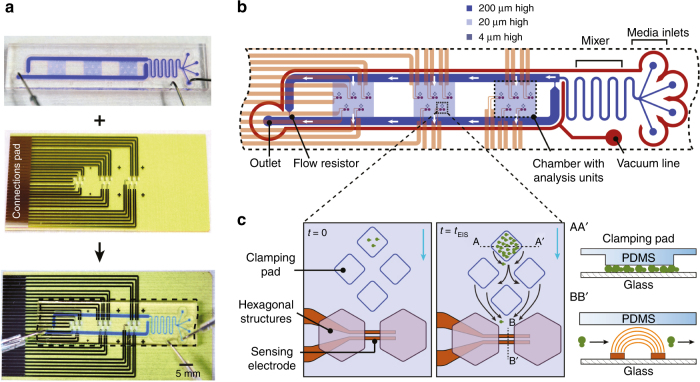
a Photographs of the PDMS layer and the glass slide with the electrodes. For device assembly, the PDMS layer was aligned with and sealed onto a glass slide. The glass appears yellowish due to the silicon nitride insulation layer. **b** Schematic illustration of the central part of the device consisting of three chambers, each having five analysis units. The 15 analysis units can be used with automated microscopy and feature integrated impedance readout. The channel for the medium is depicted in dark blue with white arrows indicating the flow direction. The vacuum channel to seal the PDMS structure against the glass and to remove bubbles in the liquid is shown in red. **c** Enlarged sketch of one analysis unit. Yeast cells are clamped under four PDMS pads and proliferate under constant media perfusion. The blue arrow indicates the flow direction of the medium, and black arrows illustrate the cell trajectories. Cross-section AAʹ illustrates that the clamping of yeast cells is between the square PDMS pads and the glass substrate. Cross-section BBʹ shows cells passing through the electric field lines of the co-planar electrodes for impedance measurements

The device consists of two parts, the PDMS layer containing the microfluidic structures and the glass slide patterned with co-planar platinum electrodes (Fig. 1a). The central part of the assembled device is schematically shown in Fig. 1b. Up to four different medium solutions can be infused through the four inlets and can be mixed in the subsequent meandered channel structure^35–37^(a feature, which has not been used in this paper) to study the effects of medium variation on the cell growth rate. The medium channels around the chamber are 1 mm wide and 200 µm high. The medium is then guided through the three microfluidic cell culture chambers with identical flow rates, as indicated by the white arrows, and is collected at the outlet. Two flow resistors (30 µm wide) are connected in parallel to the culturing chambers. They ensure a controlled, parallel, and unidirectional flow through the chambers and ensure rapid nutrient availability as well as continuous cell removal. The three chambers (4.5 mm long, 5 mm wide, and 20 µm high) allow for simultaneous culturing of three different strains under identical medium conditions.

Each chamber has five identical analysis units (Fig. 1c). An analysis unit includes four clamping pads (150 µm in diameter), two hexagonal support and guiding pillars, and one pair of sensing electrodes, placed between the guiding pillars downstream of the clamping pads. The vertical distance between the clamping pads and the glass is 4 ± 0.3 µm, which is sufficient for growing yeast cells in a monolayer under flow conditions. Once the space under the pads is completely filled, cells start to outgrow the pads. These cells are entrained in the flow around the pads and guided over the electrodes for impedance measurement. As the number of cells under the pad remains constant, the number of washed-out cells per time can be used to measure the growth rate of the cells in the chip by using impedance. The sensing electrodes are 300 µm in length, 20 µm in width, 20 µm apart, and located between two hexagonal pillar structures that focus the medium flow. The hexagonal structures also provide support to the chamber ceiling and prevent collapsing. Once the cells have passed the electrodes, they leave the chamber and are removed through the outlet. This prevents clogging of the device and allows for long-term culture.

The device accommodates a total of 15 electrode pairs, five replicates in three chambers, which are all routed to the connection pads along the side of the glass slide. The glass slide is plugged into a PCB that allows for automated sequential impedance recording through the different electrode pairs. The device with the PCB is placed in a custom-made holder mounted on a microscope stage.

### Cell culturing and cell flow in the microfluidic device

**Fig. 2:**
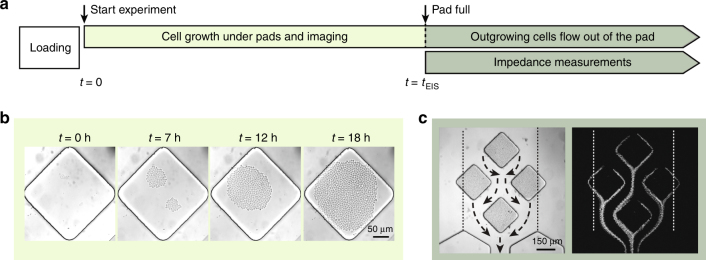
a Flow diagram of an experiment: cells were loaded into the device and allowed to grow under the pads (b). Time *t* = 0 defines the beginning of the experiment. From time *t* = *t*_EIS_ simultaneous impedance measurements of the outgrowing cells (c) and imaging of the filled pads by means of microscopy is possible. **b** Bright-field images of the cells growing between the pad and the 500-µm-thick glass slide that form a 2D monolayer. **c** Bright-field image of four filled clamping pads of an analysis unit and flow paths of cells leaving the pads as extracted from a video. The obtained cell traces indicate that all cells passed between the hexagonal guiding structures. The dotted line indicates the boundaries of the region, within which the cells were guided through the hexagonal structures and passed over the electrodes

A flow diagram of a standard experiment is shown in Fig. 2a. After loading, the cells are allowed to grow under the pads and impedance measurements of the outgrowing cells passing the electrodes are possible once the pads are filled. The setup allows for simultaneous optical imaging of the cells under the pads throughout the duration of the experiment.

Cells were loaded by pipetting the selected cell suspension (0.5 µl) into each chamber of the PDMS layer, which were then sealed with a glass slide hosting the electrodes by applying vacuum through the vacuum line. During sealing, the electrodes have to be precisely aligned with respect to the hexagonal structures in the PDMS layer. The alignment and sealing has to be quick, so that evaporation of the small volume of the cell suspension is minimized. To this end, an alignment machine that enables a simple and quick loading procedure was developed in-house (Figure S 2). The loaded volume is low enough to fill only the cell culture chamber; neither overflows of the cell suspension into neighboring chambers nor cross contamination were observed.

After sealing, the PDMS layer and glass slide were held together only through vacuum, applied to the vacuum channel that surrounds the medium microchannel inside the PDMS layer (red feature in Fig. 1b). This feature is different from what has been previously presented by Frey et al.^31^ and enables a comfortable use of the device also in laboratories lacking plasma bonding equipment. The device can be perfused for the duration of the experiment at flow rates up to 500 µl/min without leakage. The non-permanent bonding between PDMS and glass enables the facile re-use of both parts of the device. As there is a substantial risk of compound carry-over due to the absorbing nature of PDMS, only the glass slide was reused. The second function of the vacuum channel was the removal of any bubbles formed in the device after loading or during the experiment, thus ensuring robust long-term cell culturing under constant medium perfusion.

After loading, only cells below the pads were clamped. All unclamped cells outside the pads were washed away by the medium flow. The cells clamped between the PDMS and glass slide grew only in a two-dimensional (2D) plane forming a monolayer of cells, as shown in time-lapse images of one of the pads in Fig. 2b. Cell counting can be carried out by using bright-field imaging with 500-µm-thick glass slides. Clamping was found to cause no stress for the cells, as was shown previously using a Msn2 reporter^31^.

A key issue in designing the microfluidic device was to ensure that all the cells growing out of the clamping pads were guided over the sensing electrodes. Figure 2c shows a picture of the analysis units with the four pads, placed in a diamond-like configuration, and the hexagonal pillar structures further downstream. Loading of the chip leads to stochastic distribution of cells under the pads. The arrangement of the four pads in each analysis unit and their geometric area (150 µm side length) provides a sizeable number of clamped cells per analysis unit. The diamond-like shape of the pad minimizes the area of stagnant flow and prevents cell accumulation. The cell occupancy of the pads and the flow paths of the cells leaving the pads in Fig. 2c were extracted from a video. The hexagonal structures were designed to act as a funnel for collecting all cells growing out of the pads (dotted line in Fig. 2c). A finite-element model of the analysis unit evidences that the flow lines around the culturing pads pass through the hexagonal structures (Figure S 4c). It also shows the fluid velocity around the analysis unit (Figure S 4c). At the same time, the channel between the two hexagonal structures was designed sufficiently large so that all cells pass over the pair of electrodes due to the laminar-flow conditions in the chamber. A narrower channel would have entailed the risk of either clogging or of losing cells that pass outside the channel owing to the increased fluidic resistance. The five analysis units are arranged at sufficient distance within the culture chamber to completely obviate cross-talk under the given laminar-flow regime.

### Impedance measurements of cells

**Fig. 3:**
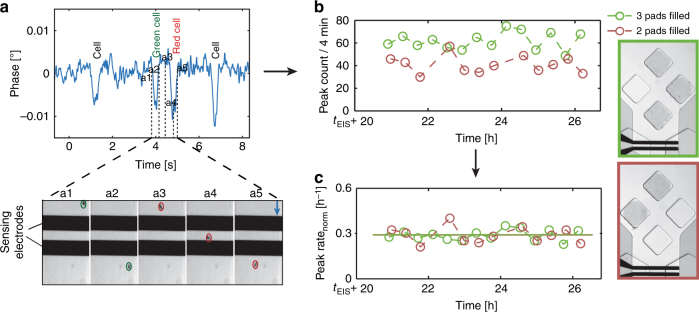
a Impedance phase signals measured at 1.5 MHz, recorded while cells pass over the electrodes.Figures a1–a5 show snapshots of events, which yielded two consecutive peaks. The first peak occurred, when the cell marked with a green circle passed over the electrode, followed by the one marked with a red circle. The blue arrow indicates the direction of the medium flow. **b** Number of peaks counted during 4-min windows through impedance measurements, plotted vs. time for two different analysis units having two and three filled pads. Each circle represents the number of peaks counted during the respective 4-min measurement window. *t*_EIS_ denotes the time interval from the beginning of the experiment until impedance values were recorded, which was 13 h in this case. **c** Peak rate_norm_ plotted for both analysis units obtained by normalizing the peak count (obtained from impedance data) for the average cell number and the recording window duration (in hours). The green and red lines by and large coincide and indicate the mean growth rate for a given cell type extracted from all the measurement windows for a given analysis unit

The electrodes downstream of the clamping pads were used to perform impedance-based counting of the cells coming from the pads. An AC voltage signal was applied to the stimulating electrode, and the current flowing through the system was converted into a voltage by using a trans-impedance amplifier. When a cell moved over the electrodes, it caused a transient variation of the intra-electrode impedance, which, in turn, produced a transient change in the recorded current and, hence, the output voltage^38,39^. A characteristic peak was observed upon passage of a cell over the electrodes in the phase component of the output voltage at 1.12 and 1.5 MHz. Impedance measurements of the cells correlated well with optical monitoring of the cells flowing over the electrodes (Fig. 3a). The impedance peaks were then used to count the cells and quantify the growth rate.

We examined the effect of the electrode area, confined by the hexagonal structures, on the impedance signal. The detection volume is defined by the width of the channel segment above the electrodes, the electrode spacing, and the height of the chamber, which is within the reach of the electric field lines. This volume defines the magnitude of the impedance change upon passage of cells. Minimization of this detection volume increased the measurable impedance signal, so that the passage of cells could be detected. With larger detection volumes, for example upon misalignment of PDMS layer and glass slide, the detection of cells was no more possible (Figure S 5).

To enable the multiple use of the glass slide hosting the electrodes, the stability of the electrodes was analyzed. A frequency sweep on all electrodes of the device evidenced low variation in phase and magnitude signal between the beginning and the end of an experiment, which indicated the robustness of the electrodes (Figure S 6a). The low variation also reflected the uniformity of the fabricated electrode pairs across the chip. Further, the signal-to-noise ratio obtained from a re-used electrode pair at identical medium conditions and measurement frequency was measured for two separate experiments (Figure S 6b). Signal peaks could be detected in both experiments, and there was no significant difference in the observed signal-to-noise ratios of the two independent experiments. These findings illustrate that a glass slide with electrodes can be used for multiple experiments.

Cells flowing over a pair of co-planar electrodes show a characteristic signature, which depends on both cell size and cell position and speed^40^. The convolution of these effects currently limits the ability of the system in distinguishing between single or budded cells, and small cell clusters. Other studies have shown that optimized geometries and setups, featuring multiple facing and focusing electrodes, provide the capability to distinguish between different cell clusters^26^ or to analyze intra-cellular features^41^. However, integration of complex sensing structures, such as sandwich structures^41^ or electrode posts perpendicular to substrate plane^42^, into the current setup would increase device complexity and compromise ease of cell loading, culturing, and imaging. The current setup meets the requirements for measuring cell growth rates and changes in those, as the passage of clustered cells over the electrodes happened very rarely, as has been confirmed with microscopy observations. This statement is supported by the fact that the growth rates determined in our system coincide with those measured by other methods. In order to compensate for not being able to measure budding cells, a correction factor (mentioned later in the paper) that is commonly used in yeast biology and includes budded cells was applied to the measured growth rates.

### Measuring growth of cell colonies

The loading process of the cells into the chamber did not produce a homogeneous cell distribution over all pad regions. Upon using low concentrations of cells in the loaded suspension in order to only clamp a few or a single cell under each pad, there is a high probability that some pads remain empty. After cell loading and starting medium perfusion, clamped cells grew and formed a 2D colony under the respective pads (Fig. 2b). Figure S 7a displays the number of cells under the pad over time, while the pad was getting filled. Once the pad was full, cells started to outgrow and were continuously removed from the colony through the medium flow around the pads. From this time on, the colony size under the pad remained stable and a counting of the outgrowing cells with respect to time could then be used as a direct measure for the growth rate of the cells under the pad.

Figure 3b presents peak counts (cells passing over the electrodes) within 4-min time windows for two different analysis units once the space under the pads was fully occupied. The analysis units featured three and two pads that had been filled with the same yeast strain. As expected, peak counts were lower for the analysis unit with two filled pads compared to that with three filled pads. To compare the measurements of the different analysis units, peak counts by the electrodes were normalized with regard to the number of cells under the respective pads. This was possible because the device allowed for simultaneous imaging of the pads during recording of the impedance signals.

Once the area under the pad was fully occupied, the variability of the cell number under the pad was low. Relative inter-pad variability in the number of cells under filled pads (in the same experiment and between experiments) was found to be approximately 8% for a given cell type (Figure S 7b). Averaged cell counts under the pads at the beginning and at the end of the impedance recording sessions were used for normalization. Further, the impedance peak count was also divided by the elapsed time of the recording window (4 min), which yielded the normalized peak rate (peak rate_norm_). The peak rate_norm_, calculated for both analysis units, was proportional to the growth rate of the cells under the respective pads. As shown in Fig. 3c, the calculated mean value of the peak rate_norm_ was found to be the same for the two analysis units (0.288 ± 0.009/h and 0.288 ± 0.015/h) in which the same strain had been cultured.

### Parallel growth rate measurements of multiple strains

The setup allows for automated switching between the sensing electrodes of different analysis units and for sequential impedance recording in a round-robin configuration during the experiments (Figure S 3). We used this feature to analyze the variations in the growth rates of different yeast strains cultured on the same device by using the impedance readout. Two strains (*vph1*∆ and wild type) were loaded and cultured in different chambers in parallel. Both strains were first grown in YPD until the pads were filled. The medium was then switched to YPD with calcium and switched back to solely YPD after approximately 10 h. The strains are known to have different growth rates when calcium is added to the medium^43^. The switching time between the two media was ~40 s at a flow rate of 10 µl/min (Figure S 4) for all analysis units, such that the recordings in all analysis units under new media conditions could be performed after less than a minute. Switching time was therefore not a limiting factor in the experiments.

**Fig. 4:**
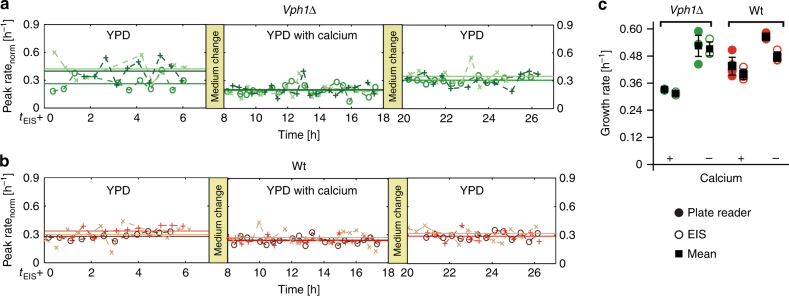
a Normalized peak rates of three replicates (represented by different symbols) of the *Vph1*∆ strain in YPD with calcium (100 mM) and in YPD without calcium. The line indicates the mean peak rate for each of the replicates. *t*_EIS_ denotes the time from the beginning of the experiment until the respective impedance values were recorded, which was 13 h in this case for both strains. **b** Normalized peak rates of the wild-type strain (replicates represented by different symbols) in YPD with calcium (100 mM) and in YPD without calcium. **c** Growth rates as obtained from the plate reader (filled circles) and the impedance device (empty circles) under different medium conditions (YPD with and without calcium) for *Vph1*∆ (green) and wild-type cells (red). Each circle indicates one replicate. Squares are means, error bars represent standard deviations

Figure 4a, b shows the normalized peak rates for the strains under different medium conditions. The average cell numbers under the pads at the beginning and at the end of the experiments for each medium condition were used for normalizing the peak rates. As expected, the mean peak rates for *vph1*∆ and the wild-type strain, grown in YPD, were similar (0.32 ± 0.02/h vs. 0.30 ± 0.01/h). When the media was switched to YPD containing calcium, the *vph1*∆ strain grew slower. The mean peak rate calculated for this condition was 0.196 ± 0.004/h for *vph1*∆ vs. 0.25 ± 0.01/h for the wild type. The cells fully recovered to their faster growth rates upon switching back to YPD without calcium (*t* = 20–26 h). We observed a higher variability in the peak rate for *vph1*∆ in YPD at the beginning of the experiment (*t* = 0–6 h) as compared to the end (*t* = 20–26 h). This finding can be attributed to the fact that the pads were initially not completely occupied, which resulted in a higher variation in the cell number under the pads and the related peak count.

The absolute growth rate of a monitored cell colony was obtained by taking into account that a fraction of cells passing over the sensing electrodes was in a budded state but yielded a single peak (Fig. 4c). As mentioned before, the current device design does not allow for differentiating between single and budded yeast cells. Therefore, the absolute growth rate was obtained after accounting for budded cells in the obtained peak rates. A budding index (percentage of budded cells) of ~0.6 (60% of all are budded) was measured for the cells used in these experiments through image analysis of the cells flowing over the electrodes. This value is comparable to the budding index value of 0.68 obtained from FACS analysis of the same cells grown under identical medium conditions in a shaking flask.

The growth rate values measured with the impedance device have also been compared to the ones obtained by using an automated plate reader for both strains under the different medium conditions. Figure 4c displays the growth rates obtained from the plate reader and obtained from the impedance device for both media: YPD with and without calcium. After adjusting the values of the impedance measurement with the budding index, the determined growth rates were comparable yet slightly lower than those obtained from the plate reader. The relative change in growth rate upon switching the medium from YPD with calcium to YPD for the *vph1*∆ strain in the plate reader and in the impedance device amounted to 58% and 63%. For the wild-type strain, the relative changes were 29% and 20%, respectively.

In summary, the developed platform enabled monitoring relative changes in growth rates of different strains in parallel upon changing the medium conditions. The change was more pronounced for *vhp1*∆ as compared to the wild type. Moreover, we showed that the replicates of the same strain yielded similar mean peak rates under the respective medium conditions, which demonstrates that the results of the impedance measurements for a given strain of cells were reproducible and consistent under identical conditions in the different analysis units. Additionally, we verified that the growth rate changes monitored in our platform were similar to those obtained with classical analysis methods.

### Optimization of the multiplexed impedance readout

The presented device features a serial readout of 15 analysis units in a sequential way. A computer-controlled switch-board was used to route the selected electrode pair to the impedance analyzer for a defined time window. During this time, the cells passing the electrodes were counted according to the obtained impedance peaks. To maximize the information that can be obtained from multiplexing several analysis units, the recording time for each analysis unit needs to be optimized. This optimization can be achieved by defining a minimal time that is required per analysis unit to obtain an accurate estimate of the cell growth rate.

**Fig. 5:**
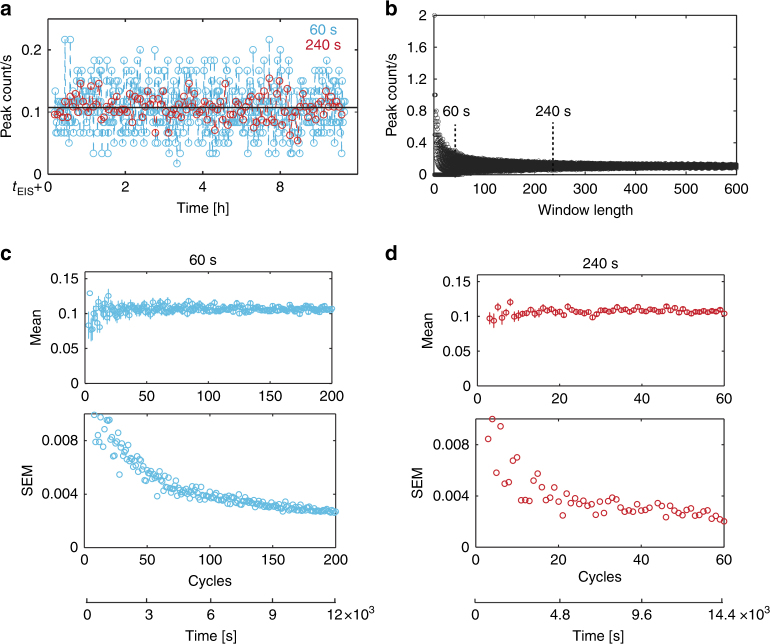
a Peak counts per second plotted for window lengths of 60 s (blue) and 240 s (red). The black line indicates the mean of the peak count for both window lengths. *t*_EIS_ denotes the time from the beginning of the experiment until the respective impedance values were recorded, which was 24 h in this case. **b** Peak counts per second for window lengths varying from 1 to 600 s. **c** Mean values with error bars indicating SEM values for 60 s window length for different numbers of cycles. Below is the same standard error of the mean (SEM) for 60 s window length for different numbers of cycles. The *x*-axis at the bottom indicates the total measurement time in seconds (cycles × window length). **d** Mean values with error bars indicating SEM values and SEM values for 240 s window length

We continuously recorded from a cell colony of a single analysis unit over 8 h to analyze the factors that may define a suitable recording time. Figure 5a shows the peak counts for time windows of 60 and 240 s. More variability in the peak count per cycle was observed for a window of 60 s as compared to 240 s. In both cases, however, the mean peak count over all measurement cycles was identical within measurement errors. Figure 5b depicts the peak count in dependence of the time window. The variability in the peak number per cycle decreased with increasing time window, as the number of peaks was averaged over a longer duration, and was found to be relatively constant for measurement windows of 300 s or more.

Larger time windows, however, reduce the sampling frequency of the analysis units during an experiment and, therefore, the temporal resolution of the obtained data (fewer red dots as compared to the blue dots in Fig. 5a). The window length and number of cycles ultimately define the obtainable measurement precision. Figure 5c, d shows the mean and standard error of the mean (SEM) for a window length of 60 and 240 s in dependence of the number of measurement cycles. As an example, a SEM of 0.004 was obtained, when an analysis unit was sampled ~85 times with a 60 s window but only ~22 times with a 240 s window. It can be inferred that for obtaining a certain precision, fewer cycles are required for recording with longer time windows from a given analysis unit. The total recording time was similar (5100 and 5280 s) for the two examples detailed above.

The number of cells passing over the electrodes is a stochastic process that can be modeled by a Poisson distribution. One feature of the Poisson model is that the precision of the estimate only depends on the total recording time, irrespective of the length of each recording interval. Consequently, it does not matter if several longer-interval or many short-interval recordings are conducted, as long as the overall recording duration is the same. It has to be noted that the window size per analysis unit should be chosen with respect to the growth rate of the cells—an analysis unit featuring slowly growing cells should be assigned a longer recording window as compared to a unit with rapidly growing cells to achieve, in both cases, good precision of the relative growth rate measurements.

### Cell imaging in the microfluidic device

The advantage of the “micro-chemostats” is that they are amenable to high-resolution imaging. We tested the imaging capabilities in the devices by using a Citrine tag endogenously fused to different yeast proteins. For high-resolution fluorescence imaging, the electrodes were patterned on a 200-µm-thick glass. The fluorescent fusion proteins localized to the vacuolar membrane (Vph1-Citrine), at the bud neck (Cdc12-Citrine) and shuffled between the cytoplasm and the nucleus in a cell-cycle-dependent manner (Whi5-Citrine). All three cell types were inoculated separately, mixed in equal ratios, and cultured in the device.

**Fig. 6:**
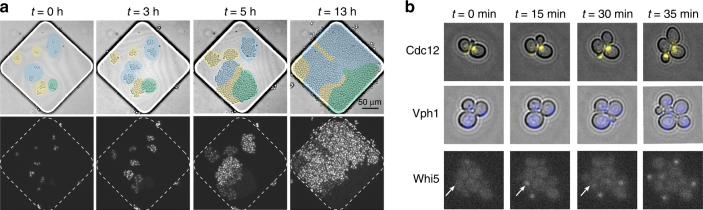
a Time-lapse images showing the growth of different cells under one of the pads (40× NA 0.75). The three strains are indicated through different colors in the bright-field images. The strains were tagged with the Citrine fluorophore at the bud neck (Cdc12; yellow), in the vacuole (Vph1; blue), and in the nucleus (Whi5; green). The pad hosts all three strains that grow, divide, and feature different fluorescence intensities of Citrine (video S 1). **b** Enlarged images of cells with the different tags. Arrows indicate the cellular distribution and transitional localization of Whi5 in the nucleus of a cell

The different cell types and their growth over time were clearly distinguishable according to their different levels of fluorophore signals (Fig. 6a). The intensity of the Citrine tag was highest for Vph1 tagged cells (~2940 copies/cell^44^), followed by Cdc12 (~680 copies/cell) and Whi5 (~120 copies/cell) tagged cells. Figure 6b shows the images of single cells featuring the different tags. The appearance of the Cdc12 marker (in yellow) at the bud neck and the localization of Whi5 in the nucleus shortly before cell division were clearly visible in the device (see also video S 1). The use of a thin glass slide makes it possible to image intracellular components and enables high-resolution microscopy comparable to that with commonly used glass slides.

## Conclusion

We described a platform enabling long-term culturing and high-resolution imaging of yeast cells, while the growth rates of populations of cells could be assessed in parallel and label free by means of impedance measurements. The platform allows for simultaneous culturing of up to three strains under different microfluidic-flow and medium conditions, and features five sets of analysis units per strain for parallelized in situ impedance analysis of the growth rate. The availability of multiple analysis units enables the user to conduct experiments in the same platform in parallel in several replications and to include all necessary control experiments, while having the capability to perform optical imaging. Importantly, the use of non-permanent vacuum-based bonding between the microfluidic structure and the glass slide hosting the electrodes allows for simple sample loading into the device and multiple usage of the glass slides.

The yeast cells grow in a monolayer under the clamping pads, they divide and develop—potentially from a single cell—to a cell colony of constant size, which is defined by the clamping pad dimensions. We validated all functions of the platform by continuously monitoring the changes in the growth rates of differently engineered yeast strains, loaded in separate culturing compartments of the device, upon changing media conditions by using the impedance readout. The observed growth rates and their changes coincide with those recorded by using standard methods. We also confirmed the quality of the optical imaging by using a set of endogenously tagged proteins that are frequently used in yeast research. This experiment shows that continuous bright-field and fluorescence imaging of the cells at sub-cellular resolution were possible with our platform.

Growth rate measurements were based on impedance and complementary to measurements performed by microscopy. Furthermore, the automated recording and switching between several impedance analysis units was completely independent of the position, temporal resolution, or field of view of the microscope used in parallel. Such a platform could serve as a useful experimental setup for nutrition and cell metabolism studies that require a continuous monitoring of growth rates in a steady and controlled environment along with tracking of fast cellular processes by means of high-resolution time-lapse imaging.

## Electronic supplementary material

Supplementary information

Video S1
