# Supplementary material for: Integrating impedance-based growth-rate monitoring into a microfluidic cell culture platform for live-cell microscopy
Source: Microsyst Nanoeng. 2018 May 24;4:8. doi: 10.1038/s41378-018-0006-5 (PMC6220194; doi:10.1038/s41378-018-0006-5)
Supplement: Supplementary file 1 — Supplementary information [file 41378_2018_6_MOESM1_ESM.docx]

Integrating impedance-based growth-rate monitoring into a microfluidic cell culture platform for live-cell microscopy

Ketki Chawla*^1^, Sebastian C. Bürgel^1^, Gregor W. Schmidt^1^, Hans-Michael Kaltenbach^2^, Fabian Rudolf^2^, Olivier Frey^1^, Andreas Hierlemann^1^

^1^ETH Zurich, Dept. of Biosystems Science and Engineering, Bio Engineering Laboratory, Basel, Switzerland

^2^ETH Zurich, Dept. of Biosystems Science and Engineering, Computational Systems Biology Group, Basel, Switzerland

Supplementary Information

**Figure S 1:** Fabrication of the device

**Figure S 2:** Loading procedure for the device

**Figure S 3:** Experimental setup

**Figure S 4:** Fluidic characterization

**Figure S 5:** Impedance signal characterization

**Figure S 6:** Electrodes characterization

**Figure S 7:** Cell numbers under the pad

**Video S 1:** Fluorescence time lapse imaging of *S.cervisae* with CDC12,Vph1 and Whi5 tagged cells.

**Fabrication process**


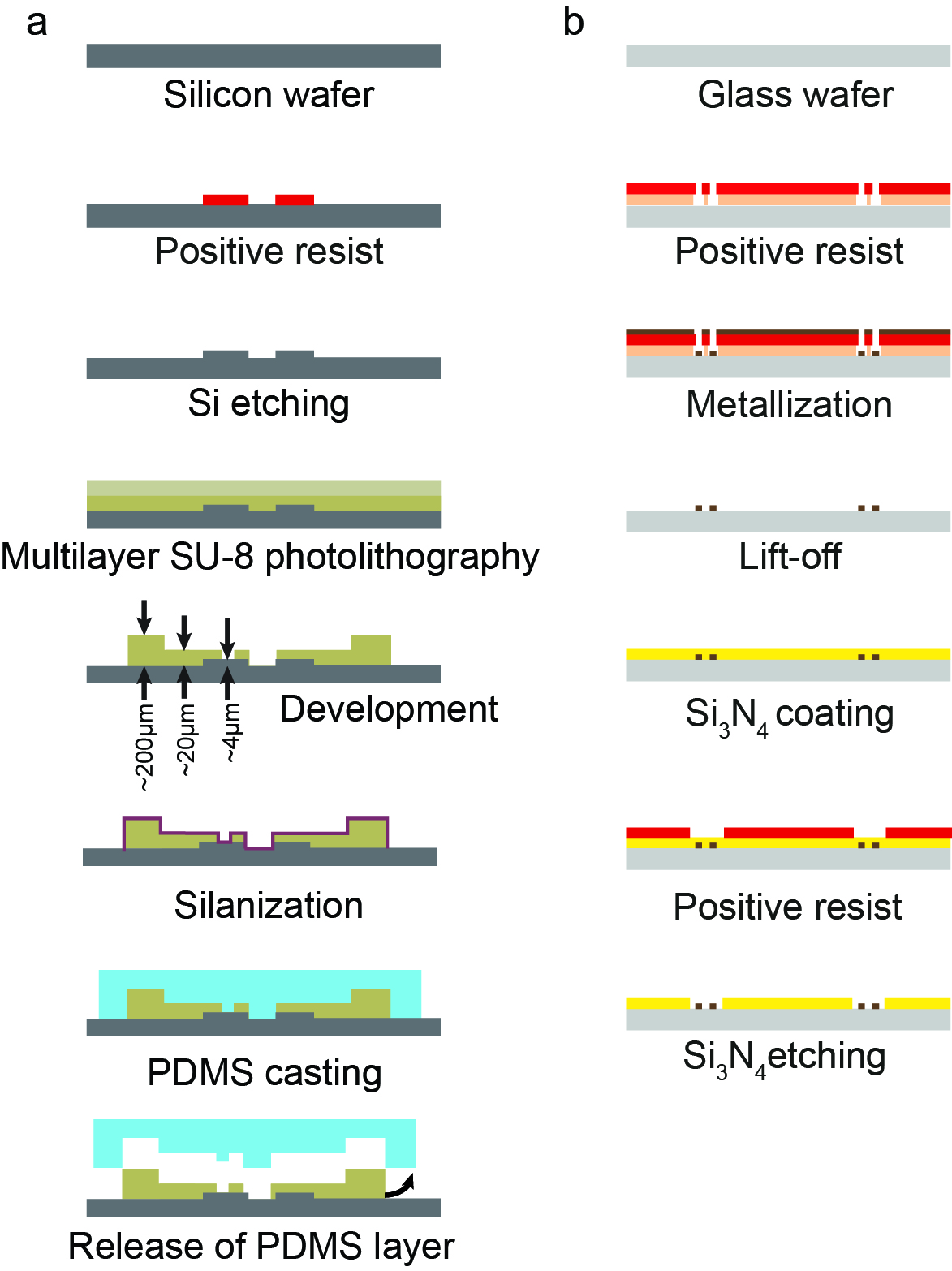


Figure S 1: Fabrication steps of the device. (a) Fabrication steps of the PDMS layer using SU-8 photolithography and PDMS molding. The silicon etching is for the clamping pads, followed by multi-layer SU-8 photolithography for the chamber and microfluidic channels. After development, the three layered SU-8 mold is silanized. Regular PDMS casting is done to obtain the PDMS layer of the device. (b)Fabrication steps for patterning the electrodes on the glass using the lift-off process. The glass is spin-coated with lift-off resist (orange) followed by a positive photoresist (red). After pattering these layers, the metal is deposited in the required areas. The metallic layer is insulated with silicon nitride except for the open electrode areas needed for measurements.

**Loading procedure**


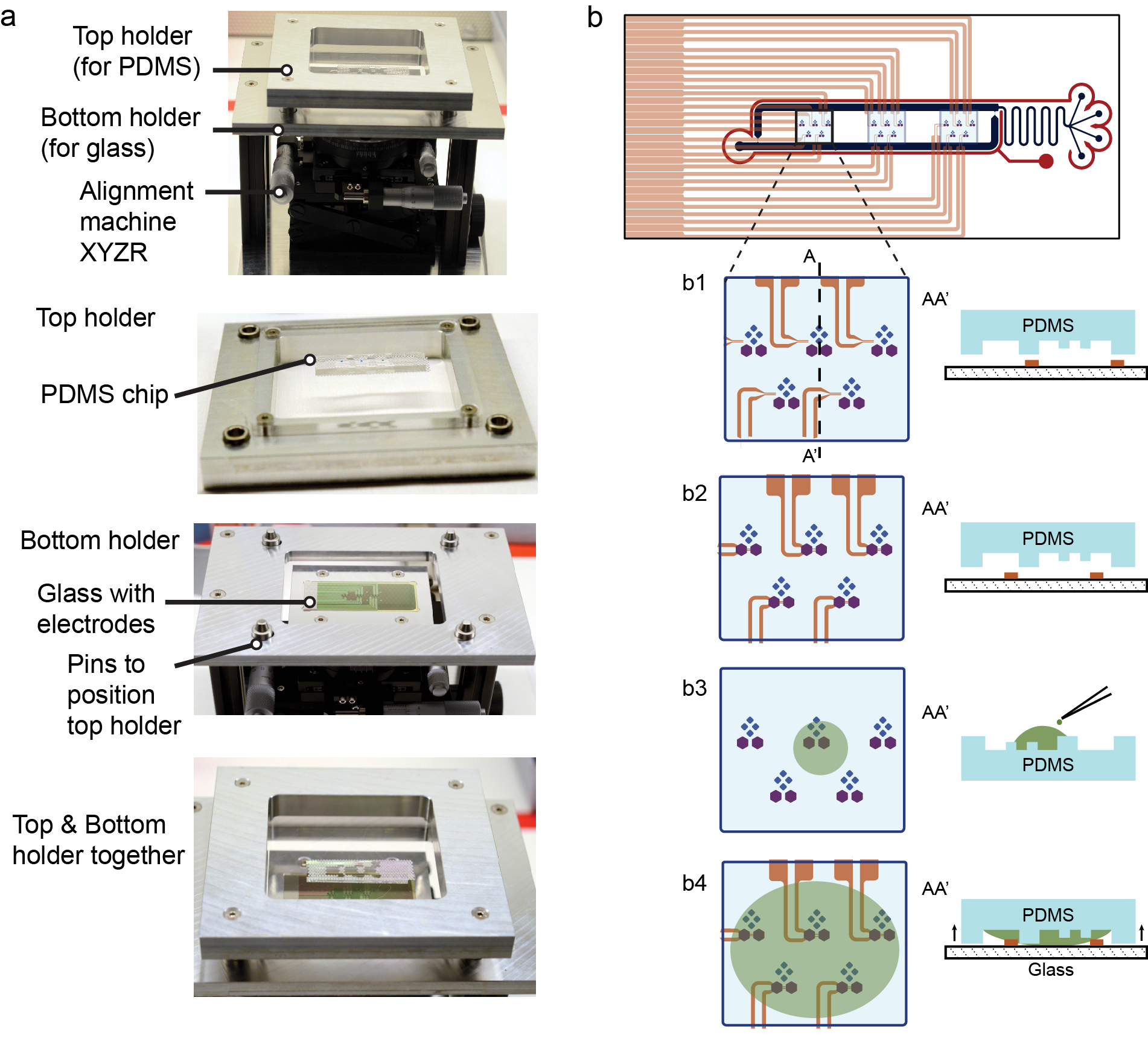


Figure S 2: (a) Picture of the alignment tool with an alignment stage to adjust the X,Y ,Z and Theta (R) directions; top holder used to place the PDMS layer; bottom holder used for the glass with electrodes. (b) Schematic showing the loading procedure. The PDMS layer and glass are brought to close proximity and aligned in X,Y,R direction (b1,b2). The top holder is removed, and cell suspension is pipetted in the chamber of the PDMS layer (b3). The top holder is placed back on the alignment machine and glass is moved upward to make contact with the PDMS layer (b4). Vacuum is then applied to the vacuum channel to seal the PDMS against the glass.

**Experimental setup**


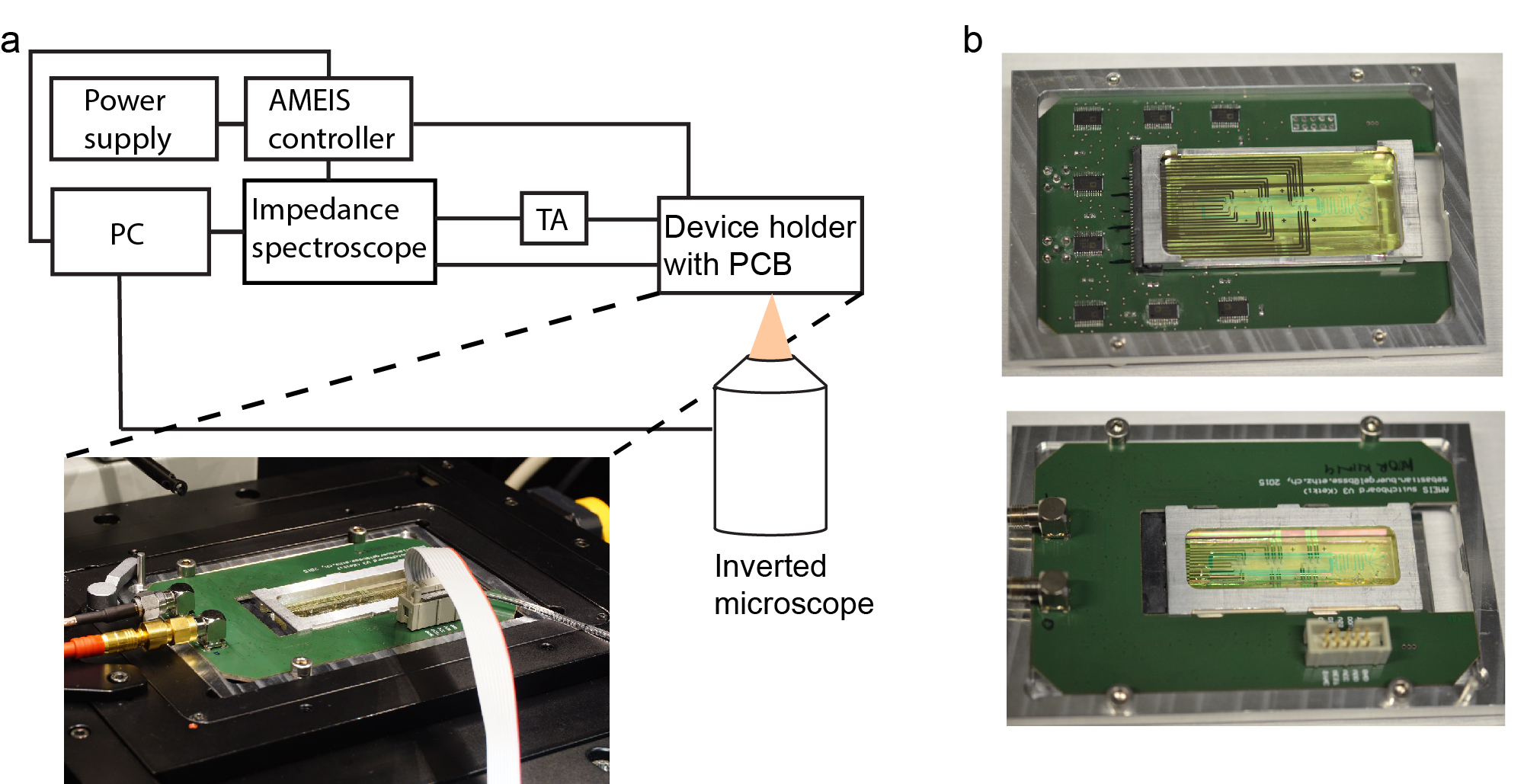


Figure S 3 : (a) Schematic of the setup. Enlarged image of the device in the holder on the stage of the microscope. The device is placed in the PCB, which is used for automated switching between the sensing electrodes of different analysis units and which routes the selected electrodes to the impedance spectroscope for measurements. The AMEIS controller communicates to the PCB the switching protocol and recording duration of the electrodes. This information has to be entered previously by the user into the custom-made software. (b) Top and bottom of the PCB with the device in the holder which can be placed in the automated stage of a microscope.

**Fluidic characterization**


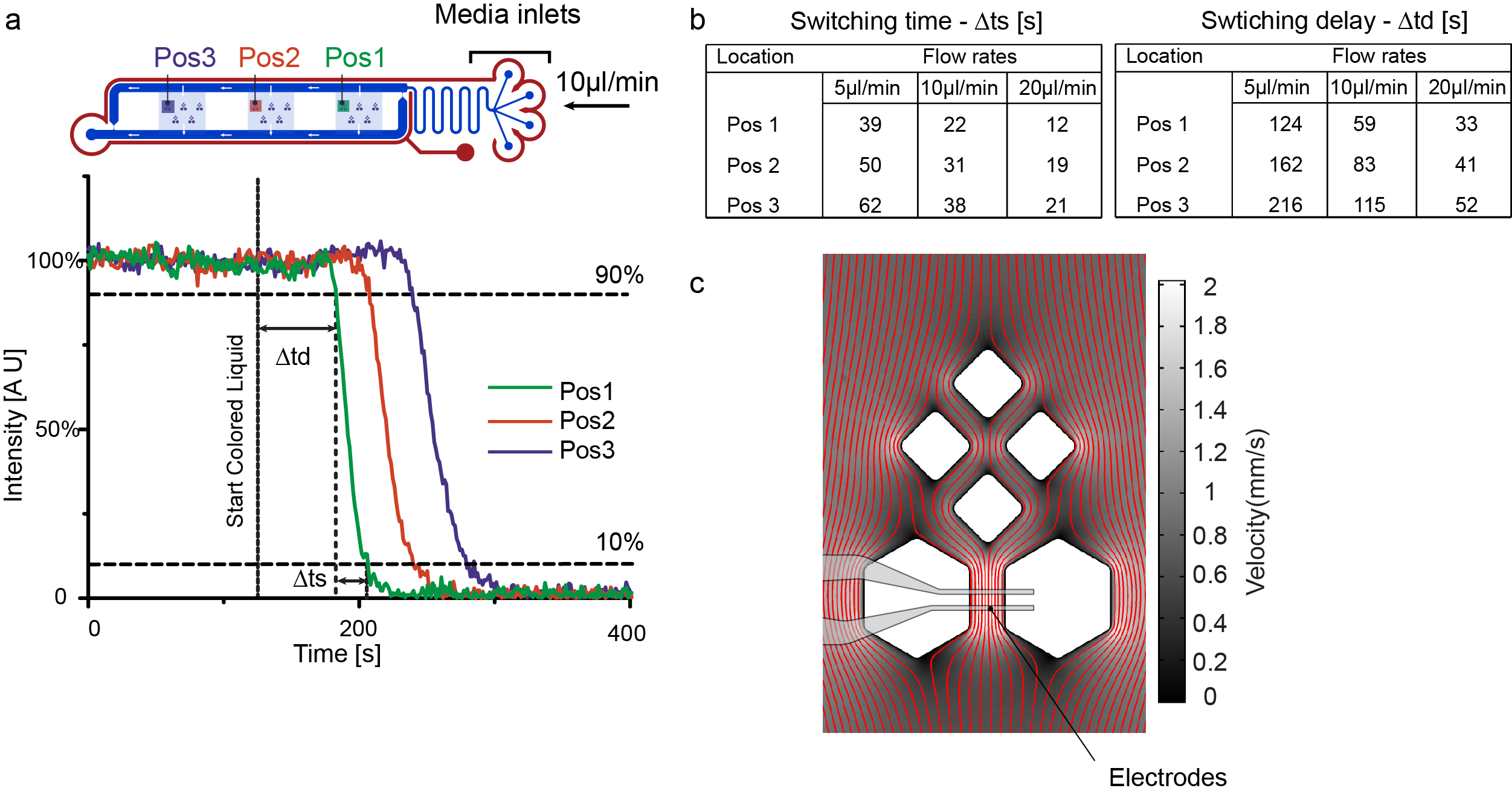


Figure S 4: (a) Medium exchange dynamics were characterized in the culture chambers by first infusing non-colored liquid (at t = 0 s) and then switching to colored liquid (t = 125 s) while performing continuous imaging. Intensity changes as a result of infused colored liquid at different positions in the device at a flow rate of 10 µl/min. Switching time (∆ts) denotes the time required to exchange the liquid at a particular position (time for the decay of the relative intensity from 90% to 10% at the respective position due to color infusion). The switching delay (∆td) is the time required for the medium to reach the analysis unit from the inlet (time from starting the infusion until a relative intensity level of 90% of the initial value is reached). (b) Table listing the switching time and delay for the three positions indicated in (a) for different flow rates.(c) Finite-element modeling of the analysis unit. The streamlines (in red) indicate the media flow inside the chip. The streamlines closer to the pads pass through the hexagonal structures, where the sensing electrodes are located. Cells growing out of the clamping pads are directed and focused towards the sensing region with the electrodes between the two hexagonal pillars at the bottom. The gray-scale color map represents fluid velocity (for an inlet flow rate 10 µl/min) throughout the analysis unit.

**Impedance characterization**


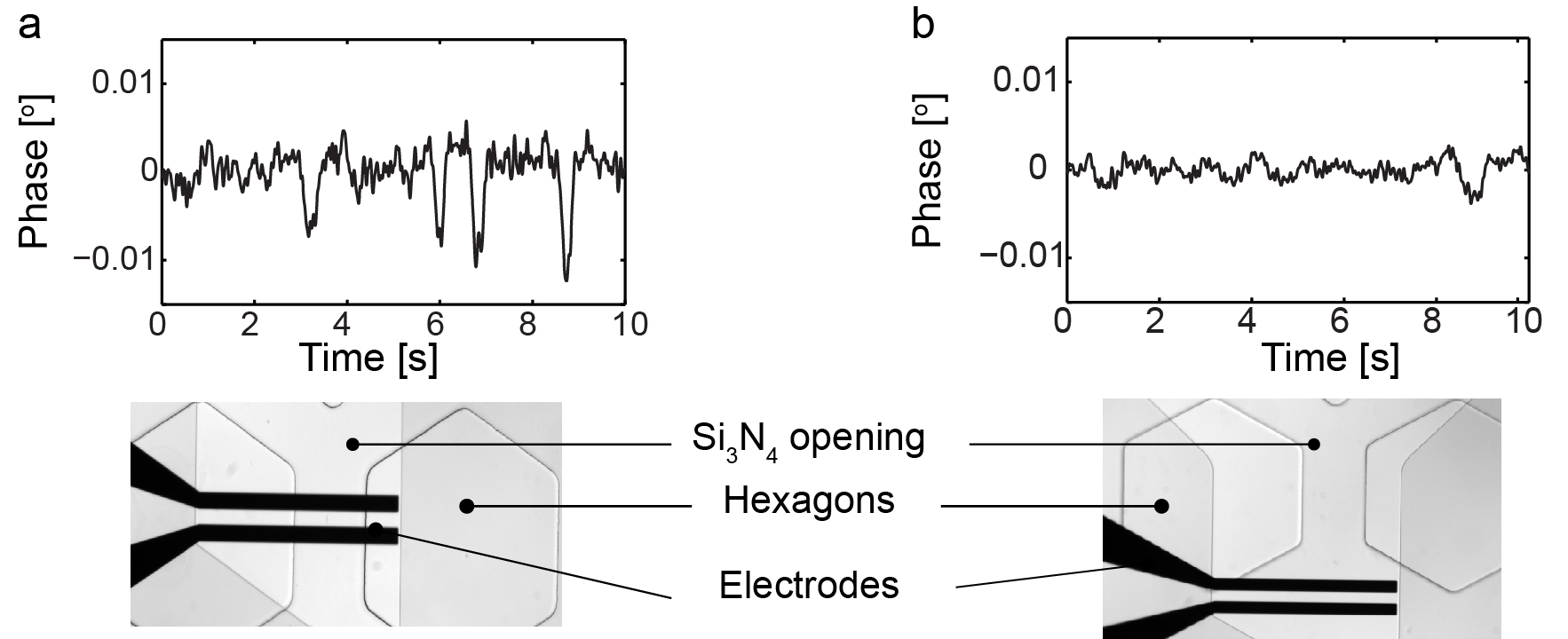


Figure S 5 : Loss of signal due to misalignment of the electrodes. (a) The figure shows the peaks when cells pass over the electrodes while those are aligned with the hexagonal structures. (b) The figure shows the loss of the signal upon misalignment, which entails an increased detection volume and less sensitive measurements.

**Electrode characterization**

**
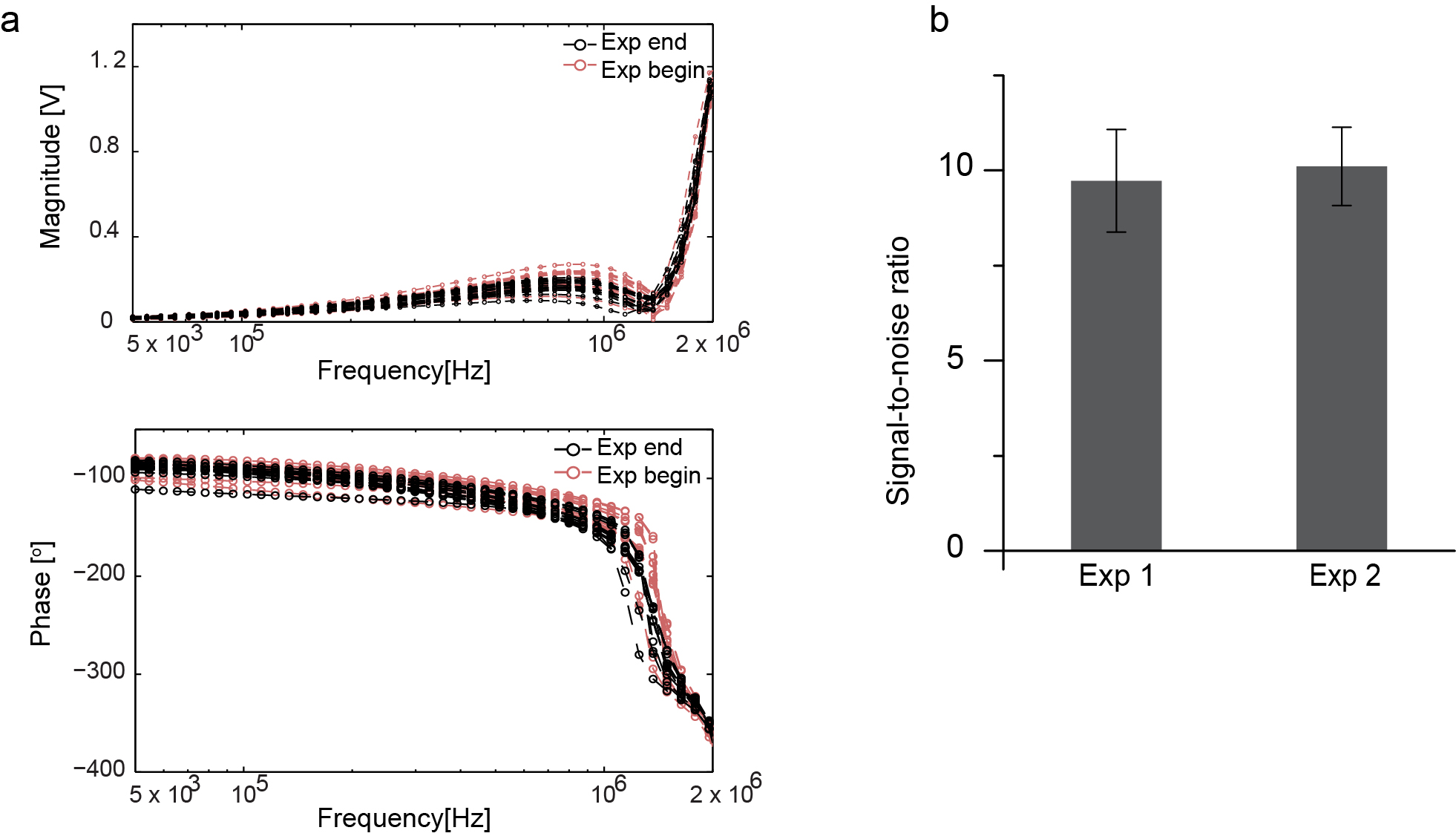
**

Figure S 6: (a) Sweep of phase and magnitude of the electrodes in a frequency range between 50kHz and 2MHz before the experiment and at the end of the experiment. (b) Mean of signal-to-noise ratio plotted for the same electrode pair, which was used for two different experiments. The mean signal-to-noise was calculated over the duration of the impedance recording under identical medium conditions and the same frequency for both experiments. The error bars represent the standard deviation. It should be noted that the glass substrate with the electrodes from experiment 1 was cleaned and aligned with a new PDMS layer and then used for experiment 2.

**Cell numbers under the pad**


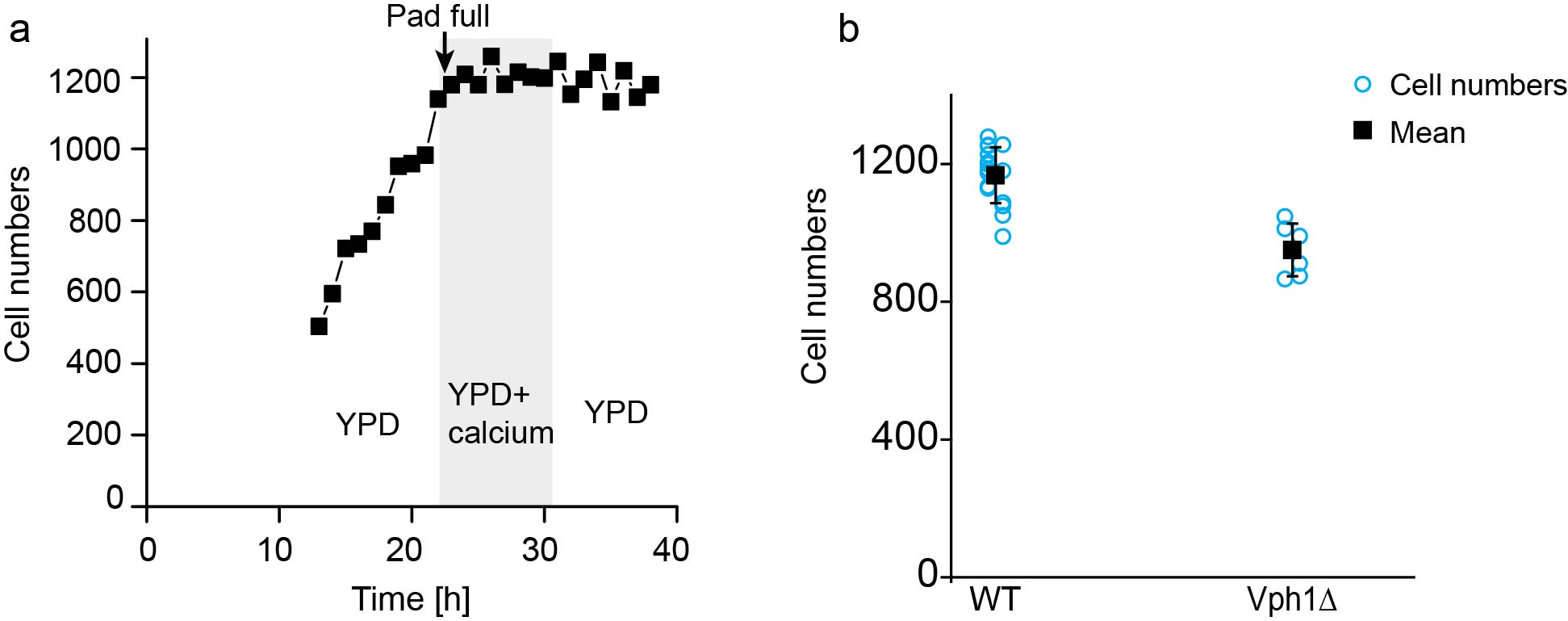


Figure S 7: (a) Cell number under a pad plotted versus time for different media conditions. Cell numbers were counted after 13 h from the beginning of the experiment. The pad was not completely occupied at this time point. The arrow indicates the point when the pad was fully occupied. The mean cell number after the pad was full (22 h - 38 h) was 1192 ± 37 (CV 3%). (b) The mean and standard deviation of cell numbers for WT and Vph1∆ cells under a full pad, calculated from two different experiments. The mean value for WT was 1167 ± 81 (CV 6%, n = 17) and was 950 ± 76 (CV 8%, n = 6) for Vph1∆. Error bars represent standard deviations.
